# Clinical pharmacist assessment of drug-related problems among intensive care unit patients in a Turkish university hospital

**DOI:** 10.1186/s12913-022-07494-5

**Published:** 2022-01-15

**Authors:** Aslınur Albayrak, Bilgen Başgut, Gülbin Aygencel Bıkmaz, Bensu Karahalil

**Affiliations:** 1 Department of Clinical Pharmacy, FacultyofPharmacy, Gazi University, Ankara, Turkey; 2 Department of Pharmacology, Faculty of Pharmacy, Baskent University, Ankara, Turkey; 3 Department of Intensive Care, Faculty of Medicine, Gazi University, Ankara, Turkey; 4 Department of Pharmaceutical Toxicology, Faculty of Pharmacy, Gazi University, Ankara, Turkey

**Keywords:** Clinical pharmacist, Drug-related problem, Intensive care unit, PCNE, Adverse drug events, Pharmaceutical care

## Abstract

**Background:**

Critically ill patients treated in the intensive care units (ICUs) often suffer from side effects and drug-related problems (DRPs) that can be life-threatening. A way to prevent DRPs and improve drug safety and efficacy is to include clinical pharmacists in the clinical team. This study aims to evaluate the classification of drug-related problems and the implementation of clinical pharmacy services by a clinical pharmacist in the ICU of a university hospital in Turkey.

**Methods:**

This study was carried out prospectively between December 2020 and July 2021 in Gazi University Medical Faculty Hospital Internal Diseases ICU. All patients hospitalized in the intensive care unit for more than 24 h were included in the study. During the study, the clinical pharmacist's interventions and other clinical services for patients were recorded. DRPs were classed according to the Pharmaceutical Care Network Europe V.8.02.

**Results:**

A total of 151 patients were included during the study period corresponding to 2264 patient-days. Patients with DRPs had a longer hospital stay and a higher mortality rate (*p* < 0.05). 108 patients had at least one DRP and the total number of DRPs was 206. There was an average of 1.36 DRPs per patient, 71.5% of patients experienced DRP and 89.22 DRPs per 1000 patient-days. A total of 35 ADEs were observed in 32 patients. ADE incidence was per 1000 patient-days 15.45. ADEs were caused by nephrotoxicity (48.57%), electrolyte disorders (17.14%), drug-induced thrombocytopenia (17.14%), liver enzyme increase (8.57%) and other causes (8.57%). Drug selection (40.29%) and dose selection (54.36%) constituted most of the causes of DRPs. Dose change was the highest percentage of planned interventions with a rate of 56.79%. Intervention was accepted at a rate of 90.8% and it was fully implemented.

**Conclusion:**

In this study, the importance of the clinical pharmacist in the determination and analysis of DRPs was emphasized. Clinical pharmacy services like the one described should be implemented widely to increase patient safety.

## Background

Critically ill patients in the intensive care units (ICUs) often suffer from some side effects and drug related problems (DRPs) that can be life-threatening [1]. Multiple organ failure and polypharmacy increase the risk of DRPs. DRP can be described as an incident or condition comprising drug treatment that actually or potentially intervenes the wished health outcomes. Most DRPs are presumable and potentially avoidable, and their frequency can be diminished with rational drug use [2, 3].

A way to prevent DRPs and improve drug safety and efficacy is to include clinical pharmacists in the clinical team. By identifying and resolving DRPs, clinical pharmacists can prevent adverse drug events from occurring [4–6]. Studies have shown that clinical pharmacists' participation in multidisciplinary teams reduces the length of stay in the ICU and mortality [7, 8]. Additionally, economic evaluations of clinical pharmacy services in the ICU consistently demonstrate the potential for significant cost savings and reduce the workload of ICU staff [5, 9].

The concept of clinical pharmacy, which was first advocated in the United States in the 1950s, was not introduced to Turkey until the 1990s [10]. Currently, there are masters, doctorate and specialization programs in Turkey. There is still insufficient number of academic staff and few clinical pharmacists routinely work in hospitals. Although there are many studies evaluating the impact of the clinical pharmacist in intensive care units [7, 11, 12], there are many differences between international health systems such as health management, local population numbers and pharmacist education. Studies related to the implementation of clinical pharmacy services, such as identifying drug-related problems, are critical for developing countries where clinical pharmacy services are emerging. This is the first comprehensive study evaluating clinical pharmacist practices in intensive care in Turkey.

This study aims to evaluate the classification of drug-related problems and the implementation of clinical pharmacy services by a clinical pharmacist in the intensive care unit of a university hospital in Turkey.

## Methods

### Study design and participants

This study was conducted prospectively between December 2020 and July 2021 in Gazi University Medical Faculty Hospital ICU of Internal Diseases Ward. There are 9 beds in the ICU of Internal Diseases Ward in the hospital.

The sample size (n) was estimated using the single population proportion formula for the finite population [13, 14]. *n* = N*X / (X + N – 1) = 200*384.16/(384.16 + 200–1) = 132, where X = Z^2^ *p*(1-p) / E^2^, and Z = 1.96 at 95% confidence level, E is the margin of error (0.05), p is the sample proportion (assigned 50% as the most conservative assumption), and N is the average population size.

All patients hospitalized in the ICU for more than 24 h were included in the study. Three doctors, one professor, one associate professor, and one assistant professor, work in the ICU. Specialist and resident physicians also work alternately. Only one clinical pharmacist worked in the study. There was no clinical pharmacist working in these clinics before the study. This study was confirmed by Gazi University Faculty of Medicine Clinical Research Ethics Committee, Ankara/Turkey and was conducted according to the Declaration of Helsinki and Good Clinical Practice (GCP). (No:700/02.11.2020).

### Data collection

During the study, the clinical pharmacist's interventions and other clinical services for patients were recorded. The data collection form was filled within 48 h of the patient's admission to the hospital. The form comprised demographics, patient history, patient diagnosis, chief complaint, history of active illness, comorbidities, family history, laboratory parameters, past medication history and daily medication list. Clinical pharmacist obtained data from electronic medical records, direct observation and visits. Additionally, clinical decision-making tools such as UpToDate®, Medscape®, Lexicomp Online®, Sanford Guide to Antimicrobial Therapy Mobile and National Kidney Foundation mobile application were used. After the suspicious DRPs were determined by the clinical pharmacist, a decision was made by discussing with the physician and nurse. PCNE Classification v.8.02 for was used to categorise DRPs by problem type, cause, planned intervention, proposed intervention, and outcome [15]. DRPs per patient were used to assess the incidence of DRP.

### Data analysis

The following variables defined the sample: age, sex, length of stay, reason for admission to the intensive care unit, mortality, and prognostic scores. Acute Physiology and Chronic Health Assessment II (APACHE II) and Sequential Organ Failure Assessment (SOFA) were obtained. Considering the parameters of admission to the intensive care unit. SPSS Software for Windows, version 20.0 (IBM, Armonk, NY) was used for the data analysis. Descriptive statistics were expressed as frequency and percentage, and the non-descriptive data were expressed as median. χ2 test and Mann-Whitney U test were used to compare categorical and continuous variables, respectively. *P*-value of < 0.05 was considered statistically significant.

## Results

During the study period, which corresponds to 2264 patient days, 151 patients were involved in our study. The causes for admission to the ICU are mainly respiratory system problems (60.9%) and sepsis (72.2%). Patients with DRPs had longer hospital stays and higher mortality rates than patients without DRPs (*p* < 0.05). APACHE II and SOFA scores did not change by DRP status (*p* > 0.05). Patient characteristics are shown in Table 1.

**Table 1:** Patient characteristics during the observation period in the intensive care unit (*n* = 151)

	Total (%)	No DRP (%)	Experienced DRP (%)	*p*
Number of patients	151	43 (28.5)	108 (71.5)	
Male	95 (62.9)	27 (62.8)	68 (68)	0.984^b^
Female	56 (37.1)	16 (37.2)	40 (37)	
Age median	69 (25–93)	68 (31–86)	69 (30–93)	0.345^a^
Length of stay, days	10 (1–97)	7 (1–40)	11 (1–97)	0.001^a^
SOFA at admission	7 (0–22)	7 (0–14)	7 (1–22)	0.715^a^
APACHE II	21 (5–40)	21 (6–33)	21 (5–40)	0.852^a^
ICU mortality	117 (77,5)	28 (65.1)	89 (82.4)	0.022^b^
Admission diagnosis – ICD 10				
J00–J99 Diseases of the respiratory system	92 (60.9)	28 (65.1)	64 (59.2)	0.581^b^
N00–N99 Diseases of the genitourinary system	11 (7.3)	2 (4.7)	9 (8.3)	0.729^d^
K00–K93 Diseases of the digestive system	12 (7.9)	1 (2.3)	11 (10.2)	0.180^d^
I00–I99 Diseases of the circulatory system	16 (10.6)	3 (7)	13 (12)	0.559^d^
A41.9 Sepsis	109 (72.2)	29 (67.4)	80 (74.1)	0.412^b^
R65.21 Septic Shock	33 (21.9)	11 (25.6)	22 (20.4)	0.484^c^
N17.9 Acute Renal Failure	46 (30.5)	11 (25.6)	35 (32.4)	0.411^b^

*SOFA* Sequential Organ Failure Assessment, *APACHE* Acute Physiology and Chronic Health Evaluation II, *ICU* Intensive Care Unit, *ICD10* International Statistical Classification of Diseases and Related Health Problems, 10th Revision, ^a^ Mann Whitney U test,, ^b^ Pearson’s χ2 test, ^c^ Continuity corrected χ2 test, ^d^ Fisher’s exact test

Table 2 presents the DRP classification. Clinical pharmacists evaluated all the patients, and 108 patients (71.5%) had at least one DRP and the total number of DRPs was 206. There was a mean of 1.36 DRPs per patient, with 71.5% of patients experienced DRP and 89.22 DRPs per 1000 patient-days. The most potential or observed problems were adverse drug events (ADEs) (77.18%). Most of the causes of DRPs are drug selection (40.29%) and dose selection (54.36%). Inappropriate drug or drug combination and herbal medicine (35.43%) had the highest percentage of drug selection. The highest percentage of planned interventions was dose changes with 56.79%. Intervention was accepted at a rate of 90.8% and it was fully implemented. Table 3 contains some examples of clinical pharmacist recommendations. As can be seen in Table 3, drug renal dose adjustment recommendations are quite high.

**Table 2:** Classification of identified drug-related problems according to the Pharmaceutical Care Network Europe Foundation

Drug-related problems (DRPs) detected	206
Patients experiencing DRP (%)	108 (71.5)
Cumulative incidence (DRP/patient)	1.36
Incidence density rate (DRP/1000 patient-days)	89.22
*Potential or manifest problems*	
P1.2 Effect of drug treatment not optimal	38 (18.44%)
P2.1 Adverse drug event occurring	159 (77.18%)
P3.1 Problem with cost-effectiveness of the treatment	5 (2.27%)
P3.2 Unnecessary drug-treatment	4 (1.94%)
*DRP causes*	
*Drug Selection*	83 (40.29%)
C1.2 Inappropriate drug (within guidelines but otherwise contra-indicated)	9 (4.36%)
C1.4 Inappropriate combination of drugs or drugs and herbal medication	73 (35.43%)
C1.5 Inappropriate duplication of therapeutic group or active ingredient	1 (0.48%)
*Dose Selection*	112 (54.36%)
C3.1 Drug dose too low	30 (14.56%)
C3.2 Drug dose too high	50 (24.27%)
C3.3 Dosage regimen not frequent enough	7 (3.39%)
C3.4 Dosage regimen too frequent	21 (10.19%)
C3.5 Dose timing instructions wrong, unclear or missing	4 (1.94%)
*Drug use process*	11 (5.33%)
C6.1 Inappropriate timing of administration and/or dosing intervals	8 (3.88%)
Other	3 (1.45%)
*The planned interventions at prescriber level*	
I1.1 Prescriber informed only	32 (15.53%)
I1.3 Intervention proposed to prescriber	174 (84.46%)
*The planned interventions at drug level*	
I3.1 Drug changed	12 (5.82%)
I3.2 Dosage changed	117 (56.79%)
I3.4 Instructions for use changed	10 (4.85%)
I3.5 Drug stopped	23 (11.16%)
I3.6 New drug started	12 (5.82%)
*Acceptance of the intervention proposals*	
A1.1 Intervention accepted and fully implemented	158 (90.80%)
A1.2 Intervention accepted, partially implemented	5 (2.87%)
A1.3 Intervention accepted but not implemented	5 (2.87%)
A2.1 Intervention not accepted: not feasible	2 (1.14%)
A2.2 Intervention not accepted: no agreement	2 (1.14%)
A3.1 Intervention proposed, acceptance unknown	2 (1.14%)
*Outcome of intervention*	
O1.1 Problem totally solved	165 (80.09%)
O2.1 Problem partially solved	17 (8.25%)
O3.2 Problem not solved, lack of cooperation of prescriber	6 (2.91%)
O3.4 No need or possibility to solve problem	18 (8.73%)

**Table 3:** Examples of clinical pharmacist recommendations*

Recommendation category	Sample pharmacist recommendation
DRP causes	
C1.2 Inappropriate drug (within guidelines but otherwise contra-indicated)	
Use of metoclopramide in patients over 65 years of age	Medication change was recommended
C1.4 Inappropriate combination of drugs or drugs and herbal medication	
Meropenem- Valproic acid	It was recommended to stop the use of meropenem
Ciprofloxacin-Enteral nutrition	It was recommended to take a 1-h break from feeding before and after ciprofloxacin administration
Phenytoin-Enteral nutrition	It was recommended to take a 1-h break from feeding before and after phenytoin administration
Clarithromycin-Midazolam	A reduction in midazolam dose was recommended
C1.5 Inappropriate duplication of therapeutic group or active ingredient	
Tiotropium bromid- ipratropium bromide	It was recommended to stop ipratropium bromide
C3.1 Drug dose too low	
Use of meropenem IV 0.5 g twice a day in a patient receiving CRRT	It was recommended to increase the dose of meropenem to 1 g twice a day
Use of Ampicillin sulbactam IV 2 g twice a day	It was recommended to increase the dose to 3 g 3 times a day
C3.2 Drug dose too high	
Use of Colistin IV 150 mg twice a day	It was recommended to reduce the dose to 110 mg twice a day in the patient with a CrCl of 50
Use of Fluconazole IV 400 mg	It was recommended to reduce the dose to 200 mg in patients with a CrCl below 50
C3.3 Dosage regimen not frequent enough	
Use of Teicoplanin every 72 h	It was recommended to be every 48 h in patients receiving CRRT
C3.4 Dosage regimen too frequent	
Teicoplanin every 48 h	It was recommended to be every 72 h in patients receiving hemodialysis
Ranitidine IV 50 mg 3 times per day	It was recommended to reduce the dose to 50 mg once a day in patients with a CrCl below 50
C3.5 Dose timing instructions wrong, unclear or missing	
Not taking additional doses after dialysis in the treatment of colistin	An additional 50 mg dose was recommended after hemodialysis
No or missing colistin loading dose	300 mg colistin loading dose was recommended
C6.1 Inappropriate timing of administration and/or dosing intervals	
Clarithromycin IV 500 mg 2 times a day	In patients receiving hemodialysis or with a CrCl less than 10, 500 mg once daily was recommended
Use of teicoplanin in patients receiving plasmapheresis	Since teicoplanin is a highly protein-bound drug, it was recommended to be given at least 4 h after plasmapheresis

*IV* Intravenous, *CrCl* Creatinine Clearance, *CRRT* Continuous Renal Replacement Therapy, *mg* milligram, *g* gram

^*^These examples were selected as important by the authors after reviewing the records of the recommendations

A total of 35 ADEs were observed in 32 patients. ADE incidence was per 1000 patient-days 15.45. ADEs were caused by nephrotoxicity (48.57%), electrolyte disorders (17.14%), drug-induced thrombocytopenia (17.14%), liver enzyme increase (8.57%) and other causes (8.57%). Piperacillin-tazobactam, linezolid, and heparin are examples of drug-induced thrombocytopenia. Colistin is the major drug responsible for nephrotoxicity. Trimethoprim-sulfamethoxazole-induced hyperkalemia is an example of electrolyte disturbances (Table 4).

**Table 4:** Drugs that cause observed adverse drug events

Patients experiencing adverse drug reactions (%)	32 (21.19)
Cumulative incidence (ADE/patient)	0.23
Incidence density rate (ADE/1000 patient-days)	15.45
Adverse drug events detected	35
*Drug-induced thrombocytopenia*	6 (17.14%)
Piperacillin tazobactam	3 (8.57%)
Linezolid	2 (5.71%)
Heparin	1 (2.85%)
*Nefrotoxicity*	17 (48.57%)
Colistin	16 (45.71)
Apixaban	1 (2.85%)
*Increase in liver enzymes*	3 (8.57%)
Tigecycline	2 (5.71%)
Meropenem	1 (2.85%)
*Electrolyte disorder (K* + *, Na* +*)*	6 (17.14%)
Trimethoprim-sulfamethoxazole induced hyperkalemia	3 (8.57%)
Heparin induced hyponatremia	1 (2.85%)
Carbamazepine induced hyponatremia	1 (2.85%)
Escitalopram-induced hyponatremia	1 (2.85%)
*Other*	3 (8.57%)
Ciprofloxacin-associated seizures	1 (2.85%)
Midazolam over-sedation	1 (2.85%)
Metformin induced lactic acidosis	1 (2.85%)

## Discussion

The study showed the significance of the clinical pharmacist's participation in the intensive care team. Most recommendations (90.8%) made by the clinical pharmacist were accepted and fully implemented by the physician.

In the study by Jiang et al. [16] the incidence of DRP per 1000 patient-days was 124.7, 112.94 in the study by Martin et al. [17] 65.1 in the study by Sakuma et al. [18]. In our study, this ratio was 89.22. These different results can be arised from various factors such as technological resources, ICU environment-specific characteristics, the method used for DRP detection, and the accepted description of DRP. Low frequencies are often seen in hospitals where a clinical pharmacist is integrated into the intensive care team [19–21].

Patients hospitalized in the ICU are exposed to polypharmacy due to their multiple comorbidities, organ dysfunction and are more prone to drug-drug interactions [5]. Most of these drugs are antimicrobials. Particularly, most of the patients hospitalized in ICUs had renal dysfunction. It is important to monitor the daily renal dose in these patients and to make dose adjustments when receiving renal replacement therapy [22, 23]. In this study, most of the DRPs were caused by inappropriate drug combinations (35.43%), overdose (24.27%) and low-dose (14.56%). In addition, dose changes (56.79%) were made in most of the interventions. Considering the other studies, drug discontinuation (23.6%) and inappropriate dose frequency (22.2%) [24], drug change (22.8%) and dose change (7.3%) [25], inappropriate drug combinations, inappropriate dose intervals (40.45%), dose change (24.09%) [17] [8] inappropriate dosing (37.3%) and drug omission (20.4%) [16] constituted most DRPs. Differences between these studies can be justified by different populations, countries and the competence of healthcare providers. In the hospital where this study was conducted, it was in close cooperation with the ICU infection team. Therefore, the duration of treatment of antimicrobials was appropriate and drug omission had not been encountered.

The incidence of ADR was 15.45 per 1000 patient-days, which is low compared to other studies; for example, Rothschild [1] detected 80 events per 1000 patient-days, Sakuma et al. [18] 37.8 events, Smithburger et al. [26] 76.2 events. Most of the adverse effects observed in our study are colistin-induced nephrotoxicity. Colistin is frequently used in ICUs for treating of multidrug-resistant (MDR) infections and as a salvage therapy [27]. For this reason, it is important to adjust the daily renal dose and pay attention to the drugs used simultaneously. Additionally, drug-induced thrombocytopenia and electrolyte disturbances were common adverse events in our study. As with other studies, most ADEs were due to antimicrobials [17, 28].

Abunahlah et al. [29] and Ertuna et al. [30] are examples of studies evaluating clinical pharmacy services in Turkey in internal medicine and geriatric services, respectively. According to studies, DRPs per patient is 1.63 ± 1.21 and 1.66 ± 0.11, which is slightly higher than our study. Similar to our study, drug selection (especially drug-drug interaction) and dose selection were the most important reasons of drug-related problems in both studies. In the study of Ertuna et al.[30], clinical pharmacy interventions were accepted at a rate of 85%, which is slightly lower than in our study.

Drug-drug interactions have a significant percentage (%35.43) in the interventions proposed in our study. Critically ill patients are more sensitive to the occurrence of drug interactions as they involve various organ failure and necessitate treatment with multiple drug combinations. Drug interactions are mainly due to administration of drugs that alter cytochrome P450 activity. [31]. The interaction of drugs that prolong the QT interval was among our interventions due to the risk of cardiotoxicity, arrhythmias and cardiac arrest.

The bioavailability of ciprofloxacin, phenytoin in the enteral feeding tube decreases by 27%-67%. The American Parenteral and Enteral Nutrition Association (ASPEN) recommend discontinuing enteral feeding 30 min before and 30 min after administration of drugs, such as ciprofloxacin whose absorption properties are affected by enteral nutrition [32]. However, many studies suggest taking a break 1–2 h before and 1–2 h after drug administration, as in our study [33].

During plasmapheresis, highly protein-bound drugs such as ceftriaxone and teicoplanin can be removed [34, 35]. In this context, the time of administration of these drugs is important. In our study, we made our recommendations on this subject according to the literature data. For example, we recommended that teicoplanin should be given at least 4 h after plasmapheresis.

Our recommendation acceptance rate (90.8%) was high considering that clinical pharmacy services are newly developing in Turkey and are comparable to the acceptance rates in other studies (61.8%-99.2%) [16, 17, 24, 36].

There are some limitations of our study. These are working in a single center, in a single intensive care unit and with a limited number of patients. Future studies should be conducted with a larger patient population with more than one clinical pharmacist evaluating the effect of the clinical pharmacist in more than one intensive care unit.

Currently, clinical pharmacy is a new field in Turkey. Our study has determined drug-related problems and shows that clinical pharmacy services should be implemented and expanded in Turkey. Additionally, our recommendations were highly accepted in our study. This is a great opportunity to show that physicians in Turkey are willing to work with clinical pharmacists.

## Conclusions

In this study, the importance of the clinical pharmacist in the determination and analysis of DRPs was emphasized. In our study, most DRPs were caused by inappropriate drug combinations and high doses. Our study showed that clinical pharmacy services are necessary and should be implemented in ICUs in Turkey. Further studies are required to show the effect of clinical pharmacist activities in ICUs.

## Data Availability

All data generated or analyzed during this study are included in this published article.

## Abbreviations

ICUs: Intensive care units
DRP: Drug related problem
APACHE II: Acute Physiology and Chronic Health Evaluation II
SOFA: Sequential Organ Failure Assessment
